# Can positional MRI predict dynamic changes in the medial plantar arch? An exploratory pilot study

**DOI:** 10.1186/s13047-016-0168-z

**Published:** 2016-09-01

**Authors:** Finn Johannsen, Philip Hansen, Sandra Stallknecht, Michael Skovdal Rathleff, Stine Hangaard, Janus Damm Nybing, Mikael Boesen

**Affiliations:** 1 Institute of Sports Medicine Copenhagen, Bispebjerg Hospital, Building 8, 1., Bispebjerg Bakke 23, Copenhagen, DK-2400, Denmark; 2 Department of Radiology, Copenhagen University Hospital Bispebjerg & Frederiksberg, Nordre Fasanvej 57, vej 4, opg. 5, Frederiksberg, DK-2000 Denmark; 3 Research Unit for General Practice in Aalborg and Department of Clinical Medicine, Aalborg University, Aalborg, Denmark; 4 Department of Occupational Therapy and Physiotherapy, Aalborg University Hospital, Aalborg, Denmark; 5 The Parker Institute, Department of Rheumatology, Copenhagen University Hospital Bispebjerg & Frederiksberg, Copenhagen, Denmark

**Keywords:** Positional MRI, Validity, Navicular bone, Medial plantar arch

## Abstract

**Background:**

Positional MRI (pMRI) allows for three-dimensional visual assessment of navicular position. In this exploratory pilot study pMRI was validated against a stretch sensor device, which measures movement of the medial plantar arch. We hypothesized that a combined pMRI measure incorporating both vertical and medial displacement of the navicular bone induced by loading would be correlated with corresponding stretch sensor measurements.

**Methods:**

10 voluntary participants were included in the study. Both pMRI and subsequent stretch sensor measurements were performed in a) supine, b) standing and c) standing position with addition of 10 % body weight during static loading of the foot. Stretch sensor measurements were also performed during barefoot walking.

**Results:**

The total change in navicular position measured by pMRI was 10.3 mm (CI: 7.0 to 13.5 mm). No further displacement occurred when adding 10 % bodyweight (mean difference: 0.7 mm (CI: −0.7 to 2.0 mm), *P* = 0.29). The total navicular displacement correlated with stretch sensor measurement under static loading conditions (Spearman’s rho = 0.66, *P* = 0.04) but not with measurements during walking (Spearman’s rho = 0.58, *P* = 0.08).

**Conclusions:**

Total navicular bone displacements determined by pMRI showed concurrent validity with stretch sensor measurements but only so under static loading conditions. Although assessment of total navicular displacement by combining concomitant vertical and medial navicular bone movements would appear advantageous compared to monoplanar measurement the combined measure did not seem to predict dynamic changes of the medial foot arch during walking, which are among several possible factors depending on different walking patterns.

**Electronic supplementary material:**

The online version of this article (doi:10.1186/s13047-016-0168-z) contains supplementary material, which is available to authorized users.

## Background

Assessment of the medial plantar arch posture is important in the clinical work-up and management of symptomatic foot disorders in both pediatric [1] and adult patients [2]. Two recent systematic reviews show that both foot posture and dynamic foot function are associated with risk of overuse injury [3, 4]. These reviews show that a pronated foot posture increases the risk of patellofemoral pain and medial tibial stress syndrome while dynamic foot function is associated with patellofemoral pain, Achilles tendinopathy, and non-specific lower limb overuse injury. Numerous clinical measures exist for characterising the foot posture, albeit there is a lack of consensus in the literature concerning the definition of foot type [5, 6]. Radiographic measurements are often used as gold standard in characterisation of foot posture, although the reproducibility of different radiographic plantar arch measures is reported to vary depending on the measurement type [1, 2]. Measurement of navicular bone height (NVH) is one such radiographic measure which appears to have high reproducibility [1, 7, 8]. However, it is fair to say that conventional radiography-based methods for measurement of foot posture have seen only little technical advancement over the last decades.

The navicular bone height (NVH) is generally considered a useful descriptor of the medial plantar arch height [9, 10]. Only few data on the validity of NVH measurement are available. A study based on electromagnetic foot motion analysis measurement of NVH in a rather large cohort (*n* = 106) found NVH to be a valid indicator of dynamic navicular bone movement as well as a “global” indicator of midfoot and rearfoot components of foot pronation or supination [11]. However, the validity of static NVH measurements in predicting dynamic foot function is a matter of debate [12–15], which could relate to the fact that the navicular bone moves not only vertically but also medially upon loading [11]. In this respect NVH measurement in isolation would be insufficient to fully describe the overall navicular bone displacement. In magnetic resonance imaging (MRI) three-dimensional (3D) sequences allow for positional assessment of a structure of interest in any anatomical plane. However, until recently MRI did not offer the possibility to provide true weight-bearing examinations. With the advent of positional MRI (pMRI) it has become possible to perform scanning under physiologic loading of the lower extremity, which allows for assessment of changes in plantar arch configuration from supine to standing position. We have previously found that both NVH and medial navicular bone position (MNP) can be determined repeatedly by pMRI, especially so in standing position [16]. Therefore, pMRI allows for measurement of the ‘total’ movement of the naviculuar bone i.e. the resultant of combined vertical and medial displacement with loading. Intuitively, it would seem likely that the 3D capabilities of MRI combined with weight-bearing could increase the value of imaging based measurements and improve understanding of the mechanical events occurring in the foot during loading. However, since the validity of static measurements in term of predicting dynamic foot function has been criticised it should be assessed whether pMRI suffers from similar limitations. The main purpose was to perform an exploratory pilot study to examine the concurrent validity of pMRI measurements of total displacement of the navicular bone against a gold standard. For this purpose we chose a recently developed stretch sensor device, which measures the movements of the medial foot arch. The latter method has been shown to be reliable for dynamic measurements during overground walking and valid compared to the static navicular drop test [17]. Measurements of loading induced change in navicular position by pMRI were compared to stretch sensor measurements performed in a) static conditions and b) dynamically during walking. We also evaluated whether any further change in navicular position occurred with addition of 10 % bodyweight in standing position.

## Methods

### Design

The study was designed as a cross-sectional exploratory pilot study and included 10 participants. Analysis of stretch sensor data was performed blinded for the results of pMRI measurements.

### Participants

Randomly recruited voluntary subjects from members of staff at the Radiology Department, Frederiksberg Hospital, Copenhagen and Institute of Sports Medicine Copenhagen, Bispebjerg Hospital gave informed consent to participate in the study, which was approved by the local ethics committee (protocol H-2-2012-151). Eligibility criteria were: age 20–50 years; no contraindications to MRI. Exclusion criteria were: self-reported foot pain or known foot disorder such as osteoarthritis, inflammatory arthritis or congenital foot deformity. The same cohort participated in a previous study in which reproducibility of pMRI measurements was assessed [16].

#### MRI procedure

**Fig. 1:**
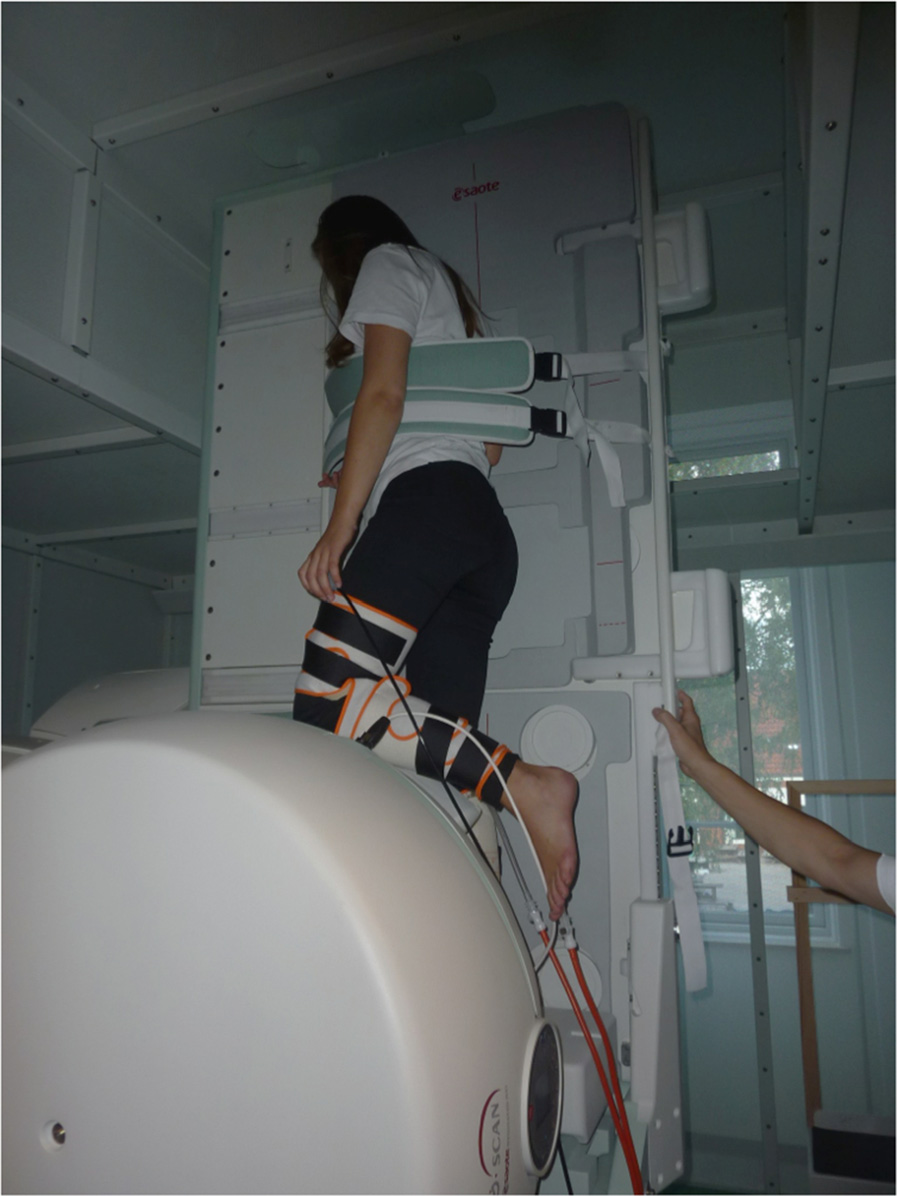
Image of a participant in weight-bearing position in the pMRI scanner. A crural pumping device is mounted to prevent symptomatic hypotension. The scanned extremity is loaded unilaterally to approximate the conditions during walking

MRI was conducted using a positional MRI-system (0,25 T G-scan, Esaote SpA, Genoa, Italy). The participants were scanned in both supine (SUP) and 90 degrees standing position (SP). The applied MRI protocol included gradient echo scout (slice thickness: 5 mm, field of view (FOV): 280x280mm, scan time 39 s) and Steady-State Free-Precession 3D (SHARC) (TE: 14 ms, TR: 28 ms, FA: 35, FOV: 230×230; Matrix: 256×256, Scan time: 6 m 54 s) sequences. As a precaution to counteract the symptomatic orthostatic hypotension or syncope during scanning a crural pneumatic pumping device was applied to stimulate the venous backflow [18]. The pMRI scanning procedure has previously been described in detail [16]. Briefly, during scanning in supine position (SUP) the talo-crural joint was positioned in a 90° angle to the long axis of the tibia. In the standing position (ST) subjects were positioned in a one-legged stance and instructed to stand with equally distributed pressure on the heel and anterior plantar sole. The non-loaded extremity rested on the housing of the scanner magnet. The foot was oriented parallel to the scanner patient table (Fig. 1). The distance from the table to the medial aspect of the foot was measured allowing the foot to be positioned similarly for stretch sensor measurements. Scanning in the standing position was repeated with addition of 10 % body weight (ST + W) carried by the participant in a backpack.

### Image analyses

**Fig. 2:**
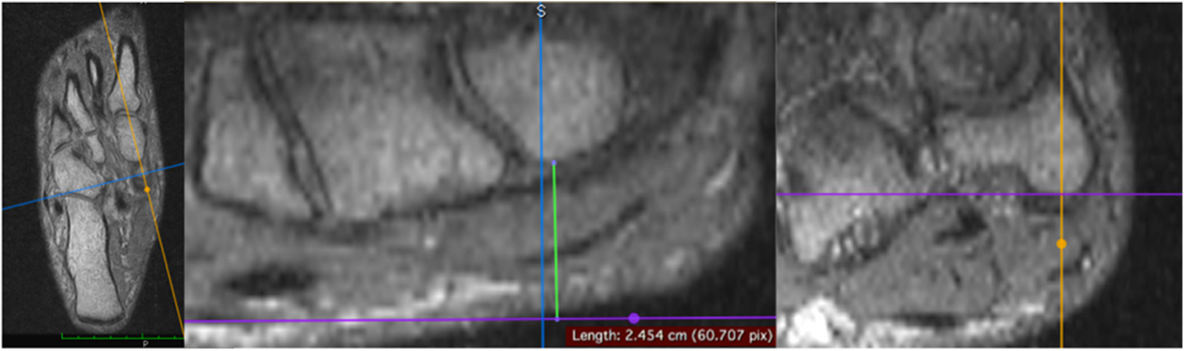
Illustration of the measurement of navicular bone height and medial navicular position in images (3D SHARC sequences) obtained by pMRI in supine and standing position respectively. By means of multiplanar reconstruction the imaging planes were adjusted in a highly standardised fashion prior to actual measurements

All image analyses were performed in a commercially available DICOM viewer (Osirix, Pixmeo SARL, Bernex, Switzerland). We have previously described the measurements of NVH and MNP in detail [16]. Briefly, owing to the 3D nature of the MRI sequences the imaging planes could be adjusted in the multiplanar reconstruction (MPR) module of the DICOM viewer in a standardised fashion before actual measurements of NVH and MNP (Fig. 2). Time consumption to perform all measurements of NVH and MNP was below 10 min per subject. One of the co-authors a 3rd year resident radiologist (PH) performed all radiological measurements reported in the present study. NVH and NMP were measured in SUP, ST and ST + W.

### Stretch sensor measurements

**Fig. 3:**
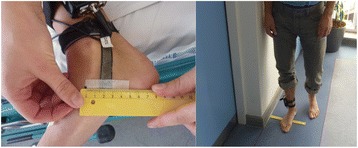
For measurement of dynamic changes in the medial plantar arch during static loading conditions and walking a stretch sensor was mounted in a standardised position spanning from behind the medial malleolus to the prominence of the navicular bone. For static measurements in standing position the foot was placed identical to the position in the pMRI unit

The newly developed strain sensor is based on a dielectric electroactive polymer material produced by Danfoss PolyPower. The material acts as an elastic capacitive material that is strainable in one direction. The sensor measures length by means of change in electrical current. It is mechanically stable, reusable, portable and resistant to perspiration from the foot. Reliability has been tested and demonstrates ICC > 0.76 for barefoot measurements [17]. The stretch sensor was attached to the skin surface as described in previous studies assessing reproducibility of the device [17, 19]: One end was attached ≈ 20 mm behind the medial malleolus and secured by a Velcro strap. The other end was attached with adhesive tape ≈ 20 mm behind the prominence of the navicular bone (Fig. 3). Measurements sampled over 15 s were performed in supine, standing and standing with 10 % extra bodyweight. In supine position a cushion under the plantar sole was used to stabilize the foot in 90 degrees ankle dorsiflexion. It was ensured that the cushion exerted only very slight pressure on the foot sole. The baseline strain on the sensor upon attachment cannot be controlled for rigorously, therefore measurements are only valid by calculating the difference between standing and supine. In standing position the foot was placed in a position identical to the position during pMRI scanning (Fig. 3). Stretch sensor measurements of the dynamic movements of the medial plantar arch were performed during barefoot walking. Walking distance was the same for all participants (20 m). Stretch sensor measurements were sampled over two contiguous walking sessions. A previous study showed that navicular drop varies from step to step [19]. Therefore, stretch sensor data were collected across two walking trials to ensure an average of more than 20 steps. The normal walking speed of each participant was established and a metronome was used to ensure this pace was consistently demonstrated throughout the gait analysis.

### Data analysis

Data were analysed using a custom-written Matlab script. Heel strike for each stance phase was manually determined using data from the accelerometer and gyroscope, which has excellent reliability and validity [19]. Afterwards, a custom written algorithm determined the maximal magnitude of navicular motion for each stance phase. The average of stretch sensor measurements over two walking sessions was used for statistical analyses. Stretch sensor measurements were performed immediately after the pMRI scanning.

### Blinding procedure

All radiologic measurements of NVH and MNP were performed by one radiologist (PH). The stretch sensor recordings were performed by another co-author (SES). Stretch sensor measurements were assessed by a third co-author (MR) blinded for subject ID and pMRI results.

### Calculation of navicular bone position changes

Change (Δ) in NVH and MNP was calculated between SUP and ST (ΔNVH_ST_; ΔMNP_ST_) and between ST and ST + W (ΔNVH_ST+W_; ΔMNP_ST+W_).

Additionally, “total” positional change of the navicular bone (ΔTPC) was calculated based on the assumption of a combined medial and caudal navicular displacement between SUP and ST. ΔTPC was expected to be better suited than NVH or MNP for direct comparison with stretch sensor measurements, as the stretch sensor measures the resultant of both vertical and medial displacement, which cannot be individually differentiated by the sensor [17]. ΔTPC was estimated by use of the equation of Pythagoras:

$$ {\mathrm{a}}^2 + {\mathrm{b}}^2 = {\mathrm{c}}^2 $$

Total positional change of the navicular bone would equal:

$$ \Delta \mathrm{TPC} = \surd \left(\Delta \mathrm{N}\mathrm{V}{\mathrm{H}}^2+\Delta \mathrm{M}\mathrm{N}{\mathrm{P}}^2\right) $$

### Statistics

ΔNVH, ΔMNP and ΔTPC are presented in mm and reported as mean with 95 % confidence intervals (CI). Data were visually assessed for normal distribution by QQ-plots and further examined by a Kolmogorov-Smirnov test. Data on navicular position by pMRI were normally distributed. Changes between scanning positions were assessed using a two-tailed paired student’s *t*-test. An alpha level of 0.05 was used. Since ΔTPC in essence has a baseline value =0 no student’s *t*-test was performed for this parameter.

ΔTPC was compared both to the delta values from the stretch sensor measurements under static loading conditions between SUP and ST and to the dynamic stretch sensor measurements during walking. Since stretch sensor data during walking were non-normally distributed a two-tailed Spearman’s Rho correlation analysis was used.

## Results

Ten healthy subjects; five females (mean age: 30 years; range: 22–39 years); mean body weight: 58; range: 49–71 kg) and five males (mean age 30 years; range: 24–38; mean body weight 75 kg; range: 63–97 kg) were included in the study. None of the participants displayed signs of orthostatic hypotension. Average values for NVH and MNP in SUP, ST and ST + W are presented as mean (range) ± SD in Table 1.

**Table 1:** Average values for navicular height and medial navicular position measurements presented as mean (range) ± SD

	Supine	Standing	Standing +10%BW
Navicular height	36.4 (25.0-47.0) ± 6.4 mm	27.7 (19.0-43.0) ± 6.7 mm	27.0 (17.0-42.0) ± 6.9 mm
Medial navicular position	45.3 (39.0-49.0) ± 3.3 mm	50.0 (45.0-52.0) ± 3.7 mm	50.4 (46.0-54.0) ± 3.1 mm

**Fig. 4:**
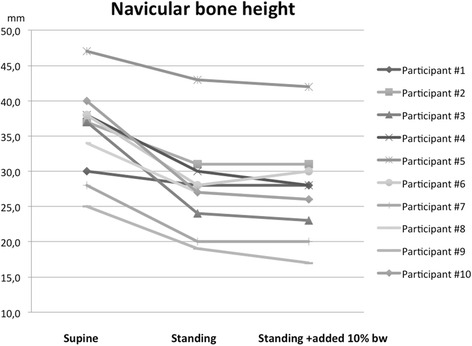
Diagram displaying navicular height for each study participant between scanning positions. There was no significant reduction in navicular height when adding 10 % bodyweight during scanning in standing position

Between SUP and ST NVH decreased (ΔNVH_ST_: 8.7 mm, CI: 6.5 to 11.0 mm, *P* < 0.001). No further decrease in NVH with addition of 10 % BW was found (ΔNVH_ST+W_ = 0.7 mm, CI: −0.2 to 1.6 mm, *P* = 0.12) (Fig. 4). Also a significant medial displacement of the navicular bone position occurred between SUP and ST (ΔMNP_ST_: 4.7 mm, CI: 1.6 to 7.8 mm, *P* < 0.01) with no additional medial displacement when adding 10 % body weight (ΔMNP_ST+W_: 0.4 mm, CI: −0.8 to 1.6 mm, *P* = 0.46).

ΔTPC (supine to standing) was 10.3 mm, CI: 7.0 to 13.5 mm. No increase in ΔTPC occurred when adding 10 % bodyweight (mean difference: 0.7 mm, −0.7-2.0 mm, *P* = 0.29).

**Fig. 5:**
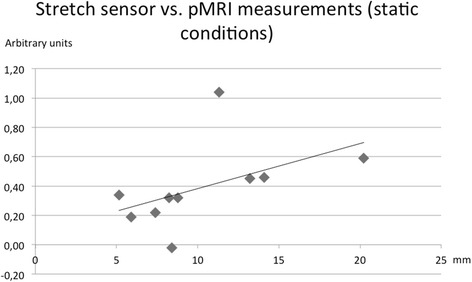
The total positional change of the navicular bone measured by pMRI and calculated by the equation by Pythagoras (ΔTPC) compared to the stretch sensor measurements under static loading conditions by means of a Spearmann’s rho correlation. ΔTPC was positively correlated with stretch sensor measurements (Spearmann’s rho = 0.66, *P* = 0.04)

ΔTPC was significantly correlated with static stretch sensor measurements (Spearman’s rho = 0.66, *P* = 0.04) (Fig. 5) but not with measurements during walking (Spearman’s rho = 0.58, *P* = 0.08).

Raw data for ΔTPC and stretch sensor measurements are provided as Additional file 1.

## Discussion

We have demonstrated that the total navicular bone movements distally and medially from supine to standing can be determined by pMRI with acceptable concurrent validity compared to stretch sensor measurements under static loading conditions. However, pMRI measurements did not correlate significantly with the dynamic measurements during barefoot walking but correlated with static stretch sensor measurement. To our knowledge this is the first study examining and validating loading induced total movement of the navicular bone measured by pMRI.

We found the mean NVH in the standing position (≈28 mm) to be slightly lower than previously reported values obtained by radiography (≈30-40 mm) as summarized by Chang et al. [20]. There are numerous possible contributors to variation in the reported values for NVH. Firstly, numerous methodologies have been applied to determine NVH across studies. One example is skin marker based measurements in which skin movement artefacts could cause some degree of error in determining the “true” position of the navicular bone [21]. Secondly, in many non-radiographic as well as some radiographic approaches both the height of the plantar sole soft tissues and the bony height of the medial plantar arch are included in the measurement [22, 23]. Soft tissue dimensions did not contribute to NVH in the present study, in which measurements solely relied on the bony architecture. Thirdly, the magnitude of foot loading in the ST position is likely to influence NVH to some degree. We opted for a single leg stance during scanning to approximate the loading conditions during walking. Previous radiographic and clinical studies of NVH have applied various loading conditions e.g. bipedal vs. unipedal standing, which are likely to influence NVH to some degree. Importantly, we observed no significant decrease in mean NVH when adding 10 % bodyweight during scanning. The total navicular position (ΔTPC) did not change with addition of 10 % extra bodyweight. Obviously, this is reassuring in terms of applying the method in longitudinal studies during which weight gain in participants may occur. The mean change in NVH from supine to standing position was ≈ 9 mm by pMRI, which is slightly higher than previous mean values reported ≈ 7 mm (range 5.3–7.4 mm) [20]. Existing data relating to MNP are scarce. We are not aware of studies assessing MNP by conventional radiography. Vinicombe et al. reported a mean medial displacement (“navicular drift”) of 7 mm measured anthropometrically from relaxed position to single limb stand [24], which slightly exceeds the mean change in MNP observed in the present study (≈5 mm). As for NVH soft tissues covering the navicular bone will contribute to anthropometric measurements while pMRI measurements of MNP relied solely on the osseous anatomy. This may explain the small discrepancy to some degree. Comparison of ΔTPC to previous radiologic studies is cumbersome since to our knowledge such data are not readily available. However, using electromagnetic foot motion analysis Cornwall & McPoil have previously reported a total navicular excursion of 7.9 (SD ± 2.5) mm resulting from combined vertical and medial displacement [11], which is somewhat less than the mean displacement (10.3 mm) found in our study. These authors performed measurements during dynamic loading conditions during walking as opposed to our static pMRI measurements. Several previous studies have stated that navicular displacement measured under static conditions does not correlate well with measurements obtained during locomotion [14, 15]. Our results are in accordance with such previous findings. Hence, the notion that measurement of the total movement of the navicular bone could improve correlation with measurements during walking could not be confirmed.

There are some limitations to the present study. Firstly, we did not assess foot posture of the included subjects prior to inclusion in the study. As such, results present are not necessarily directly comparable to other cohorts. However, it should be noted that currently there is a lack of consensus regarding cut-off values for categorizing foot posture both clinically and by radiography into low arch, normal or high arch types [2, 25, 26], which makes any pre-trial categorization troublesome. Secondly, although the NVH has been shown to be a useful descriptor of plantar arch height [7, 9] change in navicular position is obviously just one of many osseous displacements occurring with loading leading to overall changes in plantar arch posture. We chose to use the stretch sensor as a golden standard, as this method allows for dynamic measurement of the medial foot arch during walking in a fairly simple manner. However, the stretch sensor measures not only the navicular bone movements, but in principle the total movement of all the medial bones. The position of the stretch sensor 20 mm behind the malleolus and navicular tuberositas was found to be the most stable measurement position by the manufacturer, also resulted in a slightly different movement phenomena [19]. Nonetheless, the stretch sensor was found very reliable and with concurrent validity compared to anthropometric measurement by the more operator depending and time consuming Brody’s test for NVH, which is only a static test [17]. We could have chosen to use an anthropometric static measurement for the navicular position such as Brodys test, but these static tests have been shown to correlate poorly with the dynamic movements [14, 15] and we suspected that pMRI measurement of the total navicular movement could improve the correlation. A possible explanation why this was not the case could among several factors be differences in walking patterns and muscle function [27] as well as variations in walking speed [28], which might influence the dynamic results of the stretch sensor without affecting static measurements. Indeed, it has been stated that the foot is very flexible and has multiple kinematic solutions during locomotion [29]. Also, it should be mentioned that the study sample size is limited and since we have previously found some variation in pMRI measurements of navicular position especially in SUP [16] firm conclusions regarding the validity of pMRI must still be drawn with some caution. The limited sample size was considered a necessary compromise of resources. Bearing these considerations in mind, it would seem that static measurements should be reserved for describing the medial plantar arch under static loading conditions. However, this is still of obvious clinical importance as for many people the main loading of the feet is occurring in static standing position. Also for evaluation of foot surgery procedures such as plantar fascia release or other interventions to alter foot posture such as insoles static measurements remain relevant. In this regard pMRI is well suited for more elaborate measurements of navicular bone position than those feasibly obtainable by conventional radiography. Obviously, many bones other than the navicular bone move during loading and quite likely isolated measurement of navicular position is overly simple to encompass changes in medial plantar arch configuration during locomotion. We do believe that pMRI has potential to provide new insight into the more complex loading induced osseous events of the foot. Time consumption in performing measurements of navicular position by pMRI is relatively low, and in principle multiplanar displacement of any osseous component of the foot can be measured non-invasively, which is a unique feature. It seems attractive to apply pMRI in larger scale studies to examine the association between complex three-dimensional changes in foot posture and risk of injury as well as the effect of foot orthoses or surgery.

## Conclusion

In this exploratory study we have validated pMRI measurements of navicular bone displacement and find the method well suited to describe the displacement of the navicular bone under static loading conditions from supine to standing. However, although assessment of total navicular displacement by combining concomitant vertical and medial navicular position would appear advantageous compared to monoplanar measurement the combined measure did not seem to predict dynamic changes of the medial foot arch during walking, which are among several possible factors depending on different walking patterns.

## Additional file

Additional file 1: Supplementary. (XLSX 30 kb)

## Abbreviations

3D: Three-dimensional
MNP: Medial navicular position
NVH: Navicular height
pMRI: Positional MRI
ST: Standing
ST+ W: Standing + added 10 % bodyweight
SUP: Supine
TPC: Total positional change
